# The maturation zone is an important target of *Piriformospora indica* in Chinese cabbage roots

**DOI:** 10.1093/jxb/ert265

**Published:** 2013-09-04

**Authors:** Sheqin Dong, Zhihong Tian, Peng Jen Chen, Rajendran Senthil Kumar, Chin Hui Shen, Daguang Cai, Ralf Oelmüllar, Kai Wun Yeh

**Affiliations:** ^1^College of Life Science, Yangtze University, Jingzhou, Hubei 434025, China; ^2^Institute of Plant Biology, College of Life Science, National Taiwan University, Taipei, Taiwan; ^3^Institute of Molecular Phytopathology, University of Kiel, Germany; ^4^Department of General Botany and Plant Physiology, Friedrich-Schiller University, Jena, Germany

**Keywords:** Chinese cabbage, growth and biomass promotion, mutualistic symbiosis, *Piriformospora indica*, root maturation zone, subtractive EST library

## Abstract

The mutualistic symbiont *Piriformospora indica* exhibits a great potential in agriculture. The interaction between *P. indica* and Chinese cabbage (*Brassica campestris* cv. Chinensis) results in growth and biomass promotion of the host plant and in particular in root hair development. The resulting highly bushy root phenotype of colonized Chinese cabbage seedlings differs substantially from reports of other plant species, which prompted the more detailed study of this symbiosis. A large-scale expressed sequence tag (EST) data set was obtained from a double-subtractive EST library, by subtracting the cDNAs of Chinese cabbage root tissue and of *P. indica* mycelium from those of *P. indica*-colonized root tissue. The analysis revealed ~700 unique genes rooted in 141 clusters and 559 singles. A total of 66% of the sequences could be annotated in the NCBI GenBank. Genes which are stimulated by *P. indica* are involved in various types of transport, carbohydrate metabolism, auxin signalling, cell wall metabolism, and root development, including the root hair-forming phosphoinositide phosphatase 4. For 20 key genes, induction by fungal colonization was confirmed kinetically during the interaction by real-time reverse transcription–PCR. Moreover, the auxin concentration increases transiently after exposure of the roots to *P. indica*. Microscopic analyses demonstrated that the development of the root maturation zone is the major target of *P. indica* in Chinese cabbage. Taken together, the symbiotic interaction between Chinese cabbage and *P. indica* is a novel model to study root growth promotion which, in turn, is important for agriculture and plant biotechnology.

## Introduction

Over 400 million years ago, mutualistic soil fungi assisted land plants in colonizing the earth, and both partners benefited from this relationship (Bonfante and Genre, 2010). The symbiosis of root-colonizing fungi with plants plays an important role in the plant’s fitness, for nutrient acquisition or tolerance to biotic and abiotic stress (James, 1998; Borowicz, 2001). *Piriformospora indica*, a wide host root-colonizing basidiomycete of the Sebacinales (Weiss *et al.*, 2004), has been used as a model to study the mechanisms and evolution of mutualistic symbiosis (Jacobs *et al.*, 2011; Nongbri *et al.*, 2012). Decoding of the fungal genome revealed that *P. indica* may represent a missing link between a saprophytic fungus and an obligate biotrophic mutualist (Qiang *et al.*, 2012). It possesses genes for a biotrophic lifestyle, and lacks genes for nitrogen metabolism, but also shares characteristics with symbiotic fungi and obligate biotrophic pathogens (Zuccaro *et al.*, 2011). Cytological studies showed an initial biotrophic growth phase followed by a cell death-associated phase, which results in a mutually beneficial outcome (Jacobs *et al.*, 2011). *Piriformospora indica* colonizes the roots of many plant species, grows inter- and intracellularly, and forms pear-shaped spores, but does not enter the endodermis and aerial parts of the plants (Verma *et al.*, 1998, 2001; Weiss *et al.*, 2004; Sherameti *et al.*, 2005; Oelmüller *et al.*, 2009). The hallmark feature of *P. indica* is that it can be easily propagated in axenic cultures in the absence of host plants (Varma *et al.*, 1999). This attracts many researchers to use the positive effects of *P. indica* as a model system to study the molecular basis of mutualistic symbioses and symbiont-induced plant immunity (Qiang *et al.*, 2012). Genetic and biochemical analyses uncovered possible signal transduction processes induced by *P. indica* in the root cells of host plants (Shahollari *et al.*, 2005, 2007; Serfling *et al.*, 2007; Vadassery *et al.*, 2009*b*; Camehl *et al.*, 2011; Hirt *et al.*, 2011; Lee *et al.*, 2011). Furthermore, Stein *et al.* (2008) have shown that *P. indica*-induced resistance against powdery mildew requires jasmonic acid (JA), but no salicylic acid (SA) in *Arabidopsis*. Gibberellic acid (GA), JA, and SA are necessary for suppressing early immune responses in the root colonization process. *Arabidopsis* mutants impaired in GA, JA, and SA metabolism, respectively, showed elevated root immune responses and reduced colonization (Schäfer and Kogel, 2009; Schäfer *et al.*, 2009). Alteration in plant abscisic acid (ABA), GA, JA, and SA levels affected root colonization in barley (Schafer *et al.*, 2009), and changes in the GA biosynthetic pathway suppressed *P. indica*-mediated defence responses (Schäfer *et al.*, 2009). Four stages of colonization strategy have been documented in *Arabidopsis*, and characterized the broad communication between the host plant and *P. indica* (Deshmukh *et al.*, 2006; Jacobs *et al.*, 2011).

The symbiotic interaction of *P. indica* with Chinese cabbage is the authors’ area of interest (Lee *et al.*, 2011). The phenotype of colonized Chinese cabbage roots already demonstrates that the symbiotic interaction differs substantially from observations with other plant species, in particular with *Arabidopsis thaliana* and barley. Besides an overall stimulation of root and shoot growth, the massive stimulation of lateral root development results in a bushy root phenotype. Such a strong stimulation of lateral root development has not yet been described for other plant species colonized by *P. indica* (Sirrenberg *et al.*, 2007; Vadassery *et al.*, 2008; Schäfer *et al.*, 2009; Lee *et al.*, 2011; Hilbert *et al.*, 2012, 2013). This prompted the investigatation of the symbiotic interaction of *P. indica* with Chinese cabbage in more detail at the molecular and cellular level, in particular since the strong effect of the fungus on root growth and proliferation might be important for agricultural applications.

It is demonstrated that the observed phenotype is associated with a strong, transient increase of the auxin level in the roots. To identify genes which participate in growth regulation, a double-subtracted expresssed sequence tag (EST) library was constructed from colonized and control Chinese cabbage roots and the genes were annotated with bioinformatics tools. A large number of *P. indica*-induced Chinese cabbage genes associated with growth- and auxin-related functions, such as P-type/V-type H^+^-ATPase and nutrient/ion transporters, the root hair-forming phosphoinositide phosphatase 4 (RHD4), as well as cell wall loosening and synthesizing proteins involved in cell wall growth and auxin responses, were identified. Some of the genes induced during the biotrophic phase of *P. indica* colonization harmonize the auxin level with the degree of root colonization. In-depth microscopic analyses of the root structure after successful establishment of the symbiosis demonstrate that the fungus stimulates primarily growth and development of the root maturation zone, which is consistent with the identified genes in the subtractive EST library and the observed growth effects of the beneficial fungus on Chinese cabbage roots. It is concluded that *P. indica* targets primarily growth-regulating genes and processes in Chinese cabbage, which is consistent with the observed phenotype and—at least partially—different from the symbiotic interaction of the beneficial fungus with other plant species.

## Materials and methods

### Plant and fungal materials

Seeds of Chinese cabbage (*Brassica campestris* subsp. Chinesis) were donated by the MingHong Seed Company, Feng-Yuan City, Taiwan. Seeds were surface-sterilized with 75% alcohol for 10min as previously described (Lee *et al.*, 2011), then placed on Petri dishes containing half-strength Murashige and Skoog (1/2 MS) nutrient medium (Murashige and Skoog, 1962). Plates were incubated at 22 °C under continuous illumination (100 μmol m^–2^ s^–1^) for seed germination. Seven days after seed plating on 1/2 MS medium, the growing seedlings were transferred to fresh plates containing 1/2 MS medium. Up to six seedlings were used per Petri dish. One fungal disc with *P. indica* (obtained from the Deutsche Sammlung für Mikroorganismen und Zellkulturen, Braunschweig, Germany, DSM11827) or one agar disc without fungus (control) of 5mm in diameter per seedling were placed at a distance of 1cm from the roots as described in Lee *et al.* (2011). Fungal culture was maintaining on fresh solid agar medium.

### Construction of the double-subtractive EST library and analysis of EST clones

Total RNA from Chinese cabbage roots and fungal mycelium was extracted by using a protocol previously described for pine tree seedlings (Chang *et al.*, 1993). Mycelium of *P. indica* cultured on Kaefer medium (Chang *et al.*, 1993; Kumar *et al.*, 2011) for 3 weeks was used for total RNA extraction from the fungus. The mRNA was purified from the total RNA with an Oligotex mRNA Kit (Qiagene, USA), and cDNA was synthesized by using the PCR-selected TMcDNA subtraction kit following the manufacturer’s instruction (Clontech, CA, USA). The cDNA was digested with *Rsa*I and ligated to PCR adaptors. The double-subtracted hybridization was performed by using cDNAs of infected plant roots as the tester. cDNAs of non-colonized roots and of fungal mycelium were used together as drivers. According to the manufacturer’s instruction, the subtracted cDNA mixture was amplified by PCR once and the products were cloned into the pGEM-T easy vector (Promega, USA) with blue and white selection in *Escherichia coli* XL1-Blue. Two thousands white clones were randomly selected and cultured in Luria–Bertani (LB) medium at 37 °C overnight. Plasmid DNA was extracted and the insertions were sequenced. The EST sequences were assembled to obtain contigs and singletons. To annotate the clusters and singles, sequence alignment was performed by BlastX with the non-redundant protein sequence database in GenBank (NCBI), with an *E*-value threshold of E-10.

### Sequence annotation and classification

Raw sequences were trimmed using cross-matching. The insert fragments were assembled to obtain contigs and singletons. To annotate the clusters and singles, the sequences were aligned with those present in the non-redundant protein sequence database in GenBank (NCBI) by BlastX (Altschul *et al.*, 1997), with an *E*-value threshold of 1E-10. For annotation with GI numbers, all ESTs were classified and distributed onto three GO trees. The Chinese cabbage genes were named according to their closest homologues from *A. thaliana* with the prefix *BC* for *Brassica campestris*.

### RNA preparation and analysis of differential gene expression by real-time PCR

Roots of Chinese cabbage either mock treated or treated with *P. indica* were harvested 3, 5, or 7 days post-infection (dpi). A 5 μg aliquot of total RNA extracted from colonized root tissue was used to synthesize first-strand cDNA (see the protocol for cDNA synthesis, Fermentas-RevertAid First strand cDNA synthesis kit, #K1622). The quantitative reverse transcription–PCR (qRT–PCR) was performed as a two-step reaction in ABI 7500 (Applied Biosystems, USA) according to the manufacturer’s instructions. The PCR cycle condition for qRT–PCR was set as default (40 cycles): 95.0 °C for 3min, 95.0 °C for 0.03 s, 60.0 °C for 0.30 s (instruction manual, ABI Biosystems 7500 RT-PCR machine; www.appliedbiosystems.com). The relative gene expression was analysed by the 2^–ΔΔCt^ method (Livak and Schmittgen, 2001). Primer sequences are listed in Supplementary Table 10 available at *JXB* online. As an internal control, *actin* was routinely used. Data are shown as means with standard errors of three independent biological samples.

### Measurement of auxin concentrations of *P. indica*-colonized root

Seedlings of Chinese cabbage were removed from 1/2 MS plates after co-cultivation with *P. indica* or mock treatment.The roots were removed from the aerial parts and immediately ground in liquid N_2_ with a motar and pestle. Internal standards of 100ng of [^13^C]6-indole-acetic acid (IAA) were added to each sample after grinding. Tissue extraction was carried out overnight with 15ml of 80% (v/v) methanol containing 0.4mg of butylated hydroxytoluene and 2mg of ascorbate at 5 °C. The auxin levels of the extracts were analysed by gas chromatography–mass spectrometry-selected ion monitor as described previously (Lee *et al.*, 2011).

### Longitudinal microdissection of cabbage root structure

Seedlings of Chinese cabbage, 7 d after germination, were transferred to fresh 1/2 MS plates, and exposed to *P. indica* mycelium as described above, or mock treated. After 7 d, the primary roots, 3cm away from the root tip, were cut into 2cm sections for microscopic analyses. Root samples were subsequently fixed in formalin:acetic acid solution (formalin:acetic acid:50% ethanol; 1:1:18; v/w) overnight, embedded in a paraffin block, followed by longitudinal microdissection into 10–12 μm slices. The paraffin-embedded slices were washed with de-paraffinizing reagent (dimethylbenzene), and rehydrated through a serial concentration of ethanol (from 50% to 100%).

For the staining of the cell wall and the cytoplasm, the rehydrated slices were separately stained with safranin-O (100%) and aniline blue (100%). The slices were analysed and photographed under a light microscope (BH-2, Olympus, Tokyo, Japan).

### Statistical analysis

Data display means with standard errors of three independent biological samples. Two-way analysis of variance (ANOVA) was used to evaluate the differences in gene expression between colonized and non-colonized roots of *P. indica*. GraphPad Prism 5 software was used for statiscal analysis. In all graphs, the error bars indicate the standard deviation.

## Results and discussion

### Identification of P. indica target genes in Chinese cabbage roots by subtracted EST data sets

**Fig. 1.**
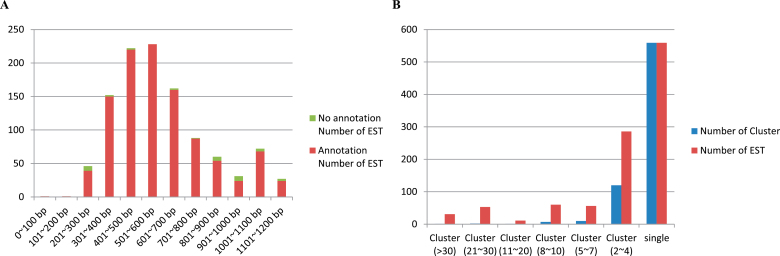
Alignment and annotation of subtractive ESTs. (A). Length distribution of subtractive ESTs. (B). Prevalence distribution of subtractive EST cluster size. The numbers in parentheses are the EST copies of clusters.

A total of 1090 clones from a double-subtractive EST library generated from *P. indica*-colonized and uncolonized root tissues were analysed and sequenced. Of these, 1056 clones with an average length of 645bp could be used for further analysis. Among them, 1054 inserts with a length of >200bp were subtractive ESTs. Their average lengths were 644bp, ranging from 200bp to 1200bp (Fig. 1A). The Phrap1054 subtractive ESTs were assembled into 487 contigs consisting of 141 clusters (divided into seven classes) and 559 singles (Fig. 1B), indicating that 52.9% of all sequenced ESTs were detected only once in the subtractive library. The complete genome sequence of Chinese cabbage is not available. Therefore, by using the sequences of the Nr and SwissProt databases as reference for alignments, 108 clusters containing 2–3 ESTs (22.5% of the total ESTs and 76.6% of all clusters) were identified. As shown in Supplementary Table 1 at *JXB* online, 12 clusters contained four ESTs (4.5% of the total ESTs and 8.5% of all clusters). The annotation uncovered that these clusters comprise ω-6 fatty acid desaturase, a ubiquitin-conjugating enzyme, a Rab family GTPase, secologanin synthase, *S*-adenosyl-l-homocystein hydrolase 1, a protein kinase, galactinol-sucrose galactosyltransferase 2, enolase, elongation factor 2, cysteine proteinase, and the 60S ribosomal protein. Eight clusters contain 5–6 ESTs (4.2% of the total ESTs and 5.6% of all clusters). These clusters were identified as arginine decarboxylase 2, glutamate synthase 1, isocitrate dehydrogenase, MLP-like protein 328, ubiquitin, glyoxalase II, and a zinc finger (C3HC4-type RING finger) family protein. Four clusters contain 7–9 ESTs (2.8% of the total ESTs and 2.8% of all clusters). They comprise cytosolic glyceraldehyde-3-phosphate dehydrogenase, cysteine proteinase, vitamin B12-independent methionine synthase isozyme, and V-type proton ATPase. Three clusters with 10 ESTs were regarded as those for the most abundant transcripts. They comprise 2.1% of the total clusters and 2.8% of all subtractive ESTs. These clusters comprise β-glucosidase 23, the epidermis-specific secreted glycoprotein, and the glutathione *S*-transferase ParC.

### Confirmation of the differential display results for a selected number of genes by qRT–PCR

After annotation and classification of the subtractive ESTs, differentially expressed genes were classified into eight categories based on biological functions (Supplementary Tables S2–S9 at *JXB* online). The majority of the proteins belonging to ‘cellular functions’ were ribosomal (31 ESTs) and cell wall metabolism-related proteins (18 ESTs; Supplementary Table 2). The majority of proteins belonging to ‘molecular functions’ were transporters (67 ESTs; Supplementary Table S3), carbohydrate metabolism proteins (43 ESTs), and transcription factors (38 ESTs). In addition, genes involved in auxin responses belonged to the largest subcategory in ‘biological processes’ (31 ESTs; Supplementary Table S4). The results implicate that genes involved in transport (67 ESTs), auxin signalling (31 ESTs), and cell wall metabolism (18 ESTs) were specifically activated or enhanced in Chinese cabbage roots during the mutualistic association with *P. indica.*

**Fig. 2.**
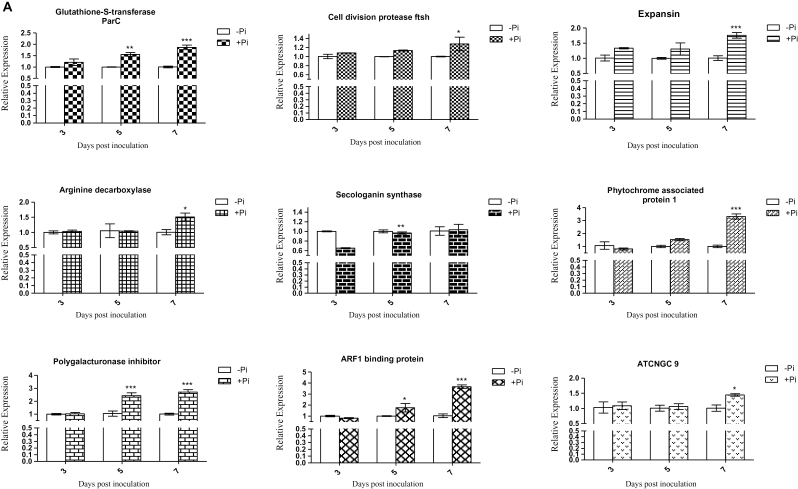

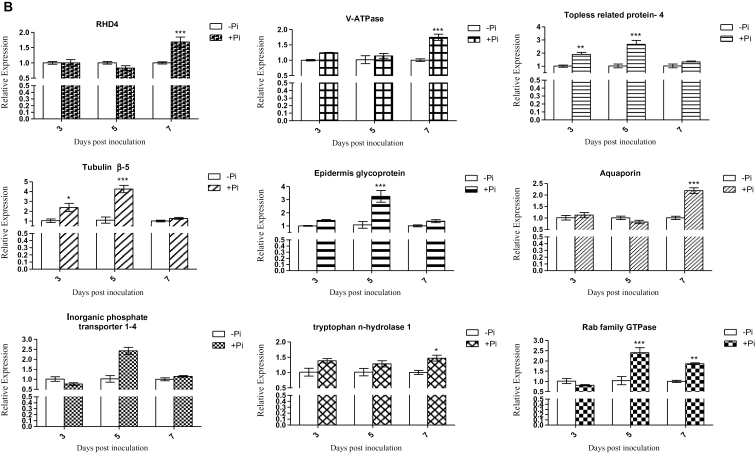

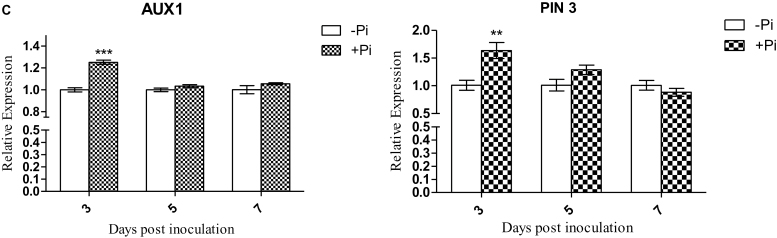
Relative quantification using real-time RT–PCR of mRNA expression of 20 genes induced by *P. indica* in Chinese cabbage root. (A–C) The expression of genes related to transporters (four), auxin biosynthesis (three), amino acid metabolism (two), cell structure and division (two), cell wall metabolism (three), stress response (two), signal transduction components (three), and root development (one) identified from ESTs was examined following *P. indica* colonization. qRT–PCR was performed to detect the level of expression of 20 genes at 3, 5, and 7 d post-inoculation of *P. indica* (+Pi). The actin gene was used as an internal control to normalize the data. The expression level of *P. indica* non-inoculated root (–Pi) was set to 1 to enable the comparison of experiments. In all graphs, the error bars indicate the standard deviation of the –ΔΔCt value, and the significant value, indicated by different numbers of asterisks was determined using two-way ANOVAs (Bonferroni post-tests), as follows: *P < 0.05; **P < 0.01; ***P < 0.001. The data are presented for three independent biological experiments.

Quantitative RT–PCR was performed for selected genes to confirm their regulation by *P. indica* in colonized Chinese cabbage roots. It has been proposed that the biotrophic root colonization phase of *P. indica* begins 2 d after inoculation (Jacobs *et al.*, 2011). Expression of putative genes which might be involved in growth promotion in response to *P. indica* and which were identified by the differential display technique was analysed 3, 5, and 7 dpi (Fig. 2A–C). These analysed genes code for transporters (four), auxin biosynthesis enzymes (three), amino acid metabolism enzymes (two), components of the cell structure and cell division (two), cell wall metabolism (three), stress response (two), signal transduction (three), and root development components (one). From the investigated time points, most of the analysed genes displayed the highest expression (two-way ANOVA, *P* < 0.001) 7 dpi. However, *BCAUX1* and *BCPIN3* expression was already high at 3 dpi, the transcript level of *BCPIN3* gradually decreased until 7 dpi, and that of *BCAUX1* had already reached the control level at 5 dpi (Fig. 2C). These data demonstrate that the identified genes are targets of *P. indica* in Chinese cabbage roots.

### Analysis of growth-related genes in *P. indica-*colonized Chinese cabbage roots

Thirty-one ESTs related to auxin biosynthesis, transport, or signalling were identified as targets of *P. indica* (Supplementary Table S4 at *JXB* online) and several of them were abundantly expressed in colonized Chinese cabbage root tissue. This includes genes for AUX/IAA repressor, ARF (auxin response factor) transcription factor, phytochrome-associated protein 1, secologanin synthase, the auxin efflux carrier proteins PIN1 and PIN3, the auxin transport protein BIG, and auxin signal compounds such as the leucine-rich repeat (LRR) protein 15 (At4g33210 homologue), the cullin-associated and E2 ubiquitin-conjugating enzyme UBC10, a 26S proteasome subunit, and a tryptophan synthase (Supplementary Table S4). Together with auxin, phytochrome coordinates *Arabidopsis* root growth and development (Salisbury *et al.*, 2007). For instance, phytochrome-associated protein 1 contributes to early lateral root emergence by transporting shoot-derived auxin to the roots (Bhalerao *et al.*, 2002) and AUX1 promotes lateral root formation by facilitating IAA distribution between sink and source tissues (Marchant *et al.*, 2002).

ARFs are key players for auxin downstream signalling events (Guilfoyle and Hagen, 2007). Auxin triggers the expression of *ARF1* for root development (Overvoorde *et al.*, 2010) and *BCARF1* was significantly more highly expressed in *P. indica*-colonized Chinese cabbage roots. The transcription factor binds to auxin response elements (Ulmasov *et al.*, 1997), consistent with the idea that *P. indica* colonization triggers root development via ARF1.

*Piriformospora indica* colonization also stimulated the expression of genes involved in auxin transport such as *BCAUX1* and *BCPIN*3 (Fig. 2B, C). *BCAUX1* and *BCPIN3* showed the highest expression already at 3 dpi. This suggests that *P. indica* induces a rapid re-distribution of auxin and/or establishes new auxin gradients within the root (Bennett *et al.*, 1996; Benkova *et al.*, 2003; Blilou *et al.*, 2005; Jones *et al.*, 2009). Benkova *et al.* (2003) showed that organ development involves dyanamic auxin gradients with maxima at the primordial tips. These gradients are mediated by cellular auxin efflux processes requiring an asymmetric localization of PIN proteins. They undergo a dynamic rearrangement, which correlates with establishment of auxin gradients and primordium development (Benkova *et al.*, 2003). Considering the microscopic data shown below, it is conceivable that *P. indica* triggers the establishment of auxin gradients to promote growth in the meristematic regions of the roots (Jiang and Feldman, 2005; Kramer and Bennett, 2006; Stoma *et al.*, 2008; Petersson *et al.*, 2009; Petrasek and Friml, 2009). Early up-regulation of *BCPIN3* after *P. indica* infection may also regulate cell division (Blilou *et al.*, 2005). This is supported by the up-regulation of the cell division genes *BCFZSH10* and *BCTUBULIN β-5* (Fig. 2A, B; Supplementary Table S9 at *JXB* online). Finally, up-regulation of *BCTIR1* and of genes involved in the ubiquitination of auxin repressors confirms increased auxin signalling. Stimulation of an auxin-responsive *B. campestris V-type ATPase* gene 7 dpi suggests that *P. indica*-induced growth promotion is associated with auxin-related cell wall acidification. According to the acid growth theory (Rayle and Cleland, 1992), auxin activates ATP-proton pumps such as the identified V-/P-type proton pump and the pyrophosphate-energized vacuolar membrane proton pump (Supplementary Table S3) to lower the extracellular pH for cell wall loosening and *de novo* synthesis (Maeshima *et al.*, 1996).

qRT–PCR analysis revealed that the *B. campestris* gene for the glutathione *S*-transferase ParC is up-regulated by *P. indica* (Fig. 2; Supplementary Table S7 at *JXB* online). ParC is involved in stress tolerance (Sakai *et al.*, 1996) and is regulated by auxin (Takahashi and Nagata, 1992; Sakai *et al.*, 1996). Thus, ParC may be involved in auxin-mediated stress tolerance during the symbiotic interaction (Takahashi and Nagata, 1992). Besides auxin-regulated genes, many other growth-related genes are up-regulated by *P. indica* (Supplementary Tables S2–S9). A total of 67 ESTs for ABC transporters, F/P-type and P/V-type proton ATPases, aquaporin, a pyrophosphate-energized vacuolar membrane proton pump, nitrate, phosphate, and glucose transporters, potassium channels, and lipid transfer proteins were identified. Besides stimulation of water and nutrient uptake, higher levels of V-/P-type proton pumps and pyrophosphate-energized vacuolar membrane proton pumps lower the extracellular pH and thus activate the enzyme activities of expansins, endoglucanases, and polygalacturonases (Supplementary Table S2) involved in cell wall expansion (Cosgrove, 2000*b*; Perrot-Rechenmann, 2010). In agreement with the growth promotion of lateral roots and root hairs and the bushy root hair phenotype (Fig. 4), *BCRHD4* was up-regulated by the endophyte in Chinese cabbage roots (Fig. 2B; Supplementary Table S5). The *Arabidopsis RHD4* gene encodes a Sac1p-like phosphoinositide phosphatase, which regulates the accumulation of phosphatidylinositol-4-phosphate at the membrane of growing root hair cells (Thole *et al.*, 2008). RHD4 is selectively recruited to RabA4b-labelled membranes which are involved in polarized expansion of root hair cells, in conjunction with the phosphoinositide kinase PI-4Kβ1 (Thole *et al.*, 2008). The database identified additional proteins with homology to *Arabidopsis* proteins involved in growth regulation, such as BCCNGC 9 and BCTopless4 (TPL4; Fig. 2A, B; Supplementary Tables S8, S9). *Arabidopsis* CNGC 9, a cyclic nucleotide-gated Ca^2+^ channel, enhances intracellular Ca^2+^ influx in response to external signals and might be involved in root development (Inoue *et al.*, 2001; Vadassery *et al.*, 2009*a*). TPLs are co-repressors recruited by AUX1 to repress auxin-responsive genes under low auxin levels (Causier *et al.*, 2012). The interplay of TPL with ARFs may be involved in establishing a local auxin homeostasis required for *P. indica*-induced growth promotion in Chinese cabbage roots (Causier *et al.*, 2012).

The identified genes involved in uptake and/or transport of organic compounds further support the idea that *P. indica* promotes nutrient (phosphate, nitrate, etc.) uptake from the environment and may therefore be important for agricultural applications. Furthermore, consistent with the strong promoting activity and the strong increase in biomass, the fungus stimulates the expression of genes involved in basic cell metabolism, such as transcription factors and ribosomal proteins. More specifically, genes involved in carbohydrate and amino acid metabolism were identified. Since the C backbone for these compounds derives from the reducing activity of photosynthesis in the leaves, it appears that the fungus also promotes root development by stimulating the transfer of reduced C from the shoot to the roots. In addition, C relocation might also be important for the heterotrophic fungus itself. The important role of sugar transporters during plant–microbe interactions has been highlighted recently by Doidy *et al.* (2012) and Smith and Smith (2012). Finally, very few genes involved in plant defence or stress responses were isolated. This clearly demonstrates that *P. indica* is accepted as a friendly partner during early phases of the symbiotic interaction.

### The auxin level is transiently up-regulated in *P. indica*-colonized roots

**Fig. 3.**
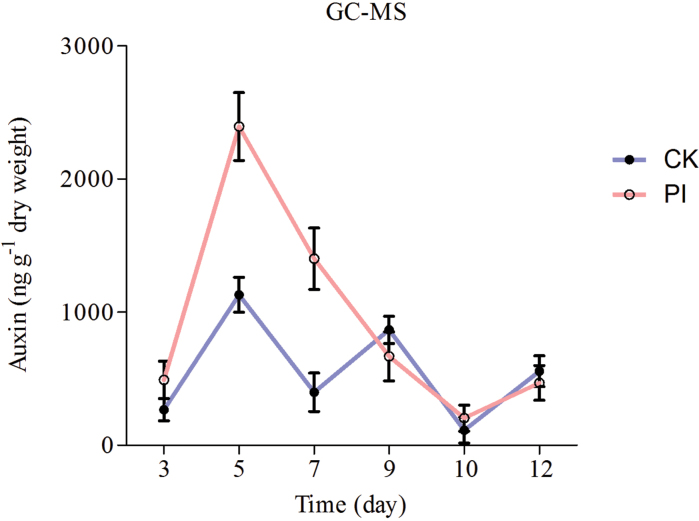
Auxin level in root tissues of *P. indica*-colonized and non-colonized cabbage roots. Root tissues were harvested from plants at 3, 7, and 10 d after colonization by *P. indica*. The data show the mean of three independent experiments.

To test whether elevated expression of auxin-related genes in *P. indica*-colonized Chinese cabbage roots is caused by elevated auxin levels, the auxin levels in the roots were determined. As shown in Fig. 3, the auxin level was low at 3 dpi, then strongly increased until 7 dpi, and fell again during later phases of the interaction. Thus, stimulation of the auxin level by the fungus in Chinese cabbage roots is consistent with the identification of auxin-regulated genes in the EST library. However, since local auxin maxima are responsible for many, if not all, auxin-mediated responses including those which are induced in plant–microbe interactions (Zhang *et al.*, 2007; Petersson *et al.*, 2009; Quint *et al.*, 2009; Seo and Park, 2009; Contesto *et al.*, 2010; Jones *et al.*, 2010; Lohmann *et al.*, 2010; Kidd *et al.*, 2011), tissue-specific and local auxin levels need to be determined, in particular in the meristematic root maturation zone.

### *P. indica* stimulates growth of the root maturation zone in Chinese cabbage

Auxin regulates the development of primary and lateral roots (Teale *et al.*, 2005; De Smet *et al.*, 2007), in which cell elongation is caused by wall loosening via cleavage of cellulosic bonds. A huge number of genes for expansins, endoglucanases, extensins, and related cell wall-modifying enzymes were identified in the library (Supplementary Table S2 at *JXB* online). qRT–PCR analysis confirmed the regulation for some of the genes (Fig. 2A). Since these genes are likely candidates for manipulation of the cell wall architecture leading to growth of the roots (Cosgrove, 2000*a*; Talke *et al.*, 2003; Aloni *et al.*, 2006; Thole *et al.*, 2008; Perrot-Rechenmann, 2010; Ljung, 2013), it was checked whether anatomical differences can be detected between *P. indica*-colonized and control roots.

**Fig. 4.**
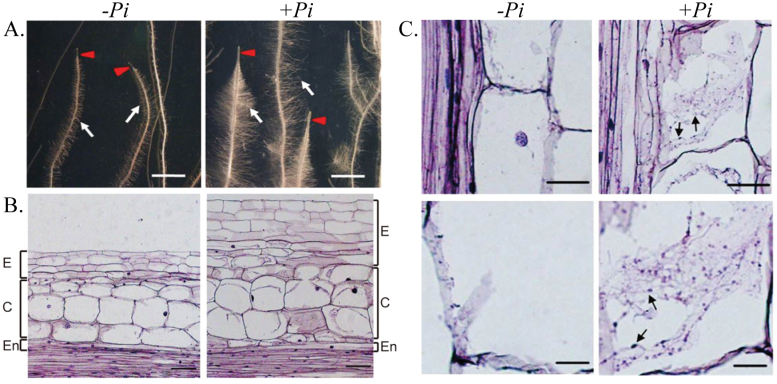
Root architecture and anatomy of Chinese cabbage colonized by *Piriformspora indica* at 7 d post-inoculation. (A) Morphology of cabbage root colonized with (+Pi) or without (–Pi) *P. indica.* Left panel: control root (–Pi) showing a poorly developed root tip (red arrow), formation of unbranched lateral roots, and less root mass (white arrow). Right panel: *P. indica*-colonized root (+Pi) showing a long and enlarged root tip (red arrow), fully developed branches of lateral root formation, and dense root biomass (white arrow). Scale bar = 2mm. (B) Longitudinal section of Chinese cabbage root stained with aniline blue. Lateral root section illustrating its structural differences with or without colonization by *P. indica*: epidermis (E), cortex (C), and endodermis (En). Left panel: control root (–Pi) showing normal characteristics of epidermal cells, cortex, and endodermis. Right panel: *P. indica*-colonized root (+Pi) showing visible expansion of the epidermis and enlargement of the cortex surface area. Scale bars = 10 μm. (C) Microscopic observation of *P. indica*-colonized root stained with aniline blue. A root longitudinal section showing hyphal penetration inside root cells and cell enlargement/expansion induced by *P. indica* colonization. Root tissues (*n*=40) used for microdissection were taken 14 d post-inoculation by *P. indica.* Left panel: control root (–Pi) cell showing no hypha and spores. Right panel: *P. indica*-colonized root (+Pi) cells of the cortex and epidermis showing intracellular hyphae and spores (arrow). Scale bars = 10 μm.

Root hair development is strongly promoted in Chinese cabbage by *P. indica* (Lee *et al.*, 2011). The lengths of the root tips and of the meristematic and elongation zones are also longer than those of uncolonized roots (Fig. 4A). On average, *P. indica* colonization resulted in an ~2-fold longer elongation zone, a 1.5-fold thicker epidermal and cortex layer, and a 1.4-fold higher biomass of the lateral roots, compared with the uncolonized control roots. Detailed anatomical analyses showed that the lower part of the root maturation zone 1 (named after Jacobs *et al.*, 2011 for *A. thaliana* roots) shows the most dramatic changes in the main root in the presence of *P. indica*. This zone contains more and enlarged cortex and epidermal cells (Fig. 4B), which results in thicker epidermal and cortex layers and ultimately larger root hairs. Hyphal penetration and chlamydospores can only be detected in and around the cortex and epidermal layers, but not in the pith region (Fig. 4C). These anatomical observations are consistent with the overall higher auxin levels. The transient increase in the auxin level may reflect transient alterations in the auxin homeostasis or local maxima after *P. indica* colonization, prior to the regulation of the above-mentioned auxin-regulated genes. Considering that exogenous application of auxin does not induce the morphological changes in Chinese cabbage roots which were observed after *P. indica* colonization (Lee *et al.*, 2011), it is unlikely that they are mediated exclusively by auxin. It is assumed that reprogramming of root development requires a battery of various fungal stimuli and host responses. However, since many of the host responses are associated with growth and proliferation, it is not surprising that auxin and auxin-induced processes play an important role in the complex interaction scenario.

Taken together, the observed morphological changes are consistent with the idea that signals from *P. indica* target primarily the meristematic elongation zone in Chinese cabbage roots to stimulate growth. It was previously demonstrated that these growth-promoting effects are mediated by auxin of plants and not fungal auxin (Lee *et al.*, 2011). These observations are in line with a recent study (Hilbert *et al.*, 2012) which demonstrated that production of indole derivatives by the fungus is not required for growth promotion of barley root. It is conceivable that signals from the fungus activate growth-stimulating programmes in roots. These programmes are activated more strongly in Chinese cabbage roots compared with other plant species, presumably because the breeding strategies optimized the activation of signalling events leading to growth and biomass production.

### The rationale of growth biomass promotion stimulated by *P. indica* colonization

**Fig. 5.**
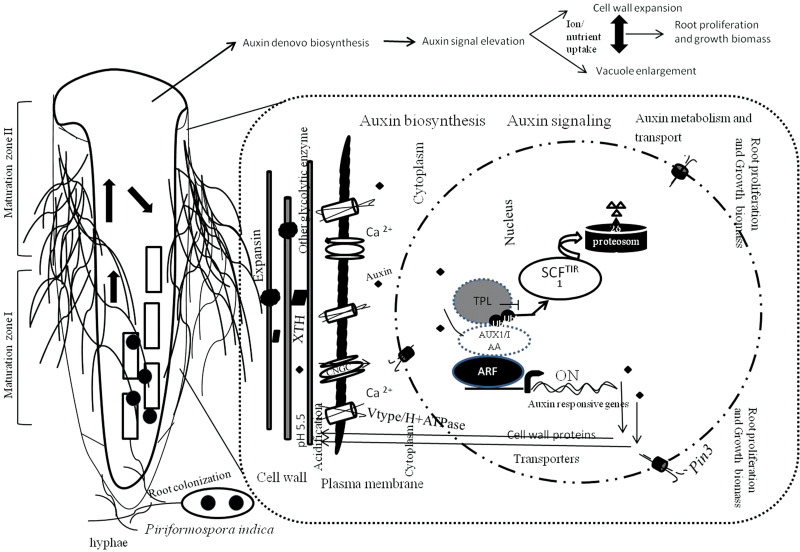
The illustration describes the proposed scheme for growth promotion effected by the mutualistic association of *P. indica*.

Plant growth results from cell division and expansion. The acid growth theory describes cell growth and is based on the concept of the space-filling function of the large vacuole and an active transport into the vacuole for maintaining a high osmotic pressure for the expansion of the vacuole and ultimately the cell (Rayle and Cleland, 1992; Cosgrove, 2000*a*; Perrot-Rechenmann, 2010). Many of the identified genes in the subtractive EST library fit into the concept of the acid growth theory. Based on the present data, a model is proposed (Fig. 5) in which Chinese cabbage responds to *P. indica* colonization by stimulating *de novo* auxin synthesis in the roots. The increased auxin levels and expression of auxin transporter genes such as *BCAUX1*, *BCPIN1*/*3*, or *BCBIG* establish the local auxin maxima required for growth and activate auxin response genes involved in growth regulation, such as genes for V/P-ATPase proton pumps and other ion transporters. *BCAUX1* overexpressor lines in *Arabidopsis* showed elevated auxin transport efficiency and this results in the promotion of plant growth (Lee *et al.*, 2011). The activation of proton pumps acidifies the intercellular space, and thus activates cell wall-loosening enzymes, such as expansins and other glycolytic enzymes. Expansin was originally discovered as a mediator of acid growth. It characteristically causes wall stress relaxation and irreversible wall extension. This process is essential for cell enlargement and is closely linked to plant cell growth. The cell wall expansion is associated with the stimulation of nutrient/ion uptake, as demonstrated by the higher expression of genes for those transporters.

In summary, the data presented in this study provide evidence that the genes identified from the EST library revealed differential expression between *P. indica*-colonized and control root tissues. Among them are auxin-related (31 ESTs), transporter (67 ESTs), and cell wall-loosening/synthesizing genes (17 ESTs), which may explain the growth promotion mechanism. Moreover, the alteration in the structure of the root anatomy and the elevated auxin level in colonized roots provide supporting evidence for the role of these genes for growth promotion and root proliferation. It has long been known that root development is controlled by local auxin maxima and a highly sophisticated auxin homeostasis. It is conceivable that this homeostasis is modified by the fungus in favour of growth promotion. More interestingly, the root maturation zone shows the most dramatic changes in response to *P. indica*, and colonized roots contain more and enlarged cortex and epidermal cells. It is proposed that realization of the *P. indica*-induced reprogramming of Chinese cabbage root development is initiated in this area. The present systematic analysis of EST data, auxin levels, and anatomical changes allows the study of growth regulation in response to fungal signals. Moreover, the identified *P. indica* target genes may allow the manipulation of plant performance in agriculture.

## Supplementary data

Supplementary data are available at *JXB* online.

Table S1. Assembled clusters that contains more than four ESTs.

Table S2. Selected examples of ESTs for genes related to cell wall metabolism.

Table S3. Selected examples of ESTs for genes related to transport.

Table S4. Selected examples of ESTs for genes related to phytohormone biosynthesis and response.

Table S5. Selected examples of ESTs for genes related to root development.

Table S6. Selected examples of ESTs for genes related to amino acid metabolism.

Table S7. Selected examples of ESTs for genes related to stress response.

Table S8. Selected examples of ESTs for genes related to signal transduction.

Table S9. Selected examples of ESTs for genes related to cell structure and division.

Table S10. List of primers.

Supplementary Data
