# Supplementary material for: The maturation zone is an important target of Piriformospora indica in Chinese cabbage roots
Source: J Exp Bot. 2013 Sep 4;64(14):4529–40. doi: 10.1093/jxb/ert265 (PMC3808330; doi:10.1093/jxb/ert265)
Supplement: Supplementary Data [file supp_64_14_4529__index.html]

The maturation zone is an important target of Piriformospora indica in Chinese cabbage roots — The maturation zone is an important target of Piriformospora indica in Chinese cabbage roots — Supplementary Data 

# The maturation zone is an important target of *Piriformospora indica* in Chinese cabbage roots

## Supplementary Data

Data files

**Files in this Data Supplement:**

- Supplementary Data - Supplementary Data
