# Supplementary material for: The maturation zone is an important target of Piriformospora indica in Chinese cabbage roots
Source: J Exp Bot. 2013 Sep 4;64(14):4529–40. doi: 10.1093/jxb/ert265 (PMC3808330; doi:10.1093/jxb/ert265)
Supplement: Supplementary Data [file supp_ert265_jexbot099531_file001.pdf]

Supplementary Table S1. Assembled clusters that contain more than four ESTs

Gene annotation	Reference organism	GI number	E-value	ESTs
β-glucosidase 23	*A. thaliana*	75313794	1E-91	10
epidermis-specific secreted glycoprotein	*D. carota*	34921531	3E-52	10
glutathione S-transferase parC	*N. tabacum*	1346215	5E-44	10
cytosolic glyceraldehyde-3-phosphate dehydrogenase	*S. alba*	120675	2E-127	9
cysteine proteinase	*A. thaliana*	30315954	3E-64	7
Vitamin-B12-independent methionine synthase isozyme	*A. thaliana*	8134566	5E-77	7
V-type proton ATPase	*A. thaliana*	15233060	6E-135	7
arginine decarboxylase 2	*A. thaliana*	12229960	5E-118	6
glutamate synthase 1	*A. thaliana*	300680981	2E-37	6
isocitrate dehydrogenase	*G. max*	1708401	2E-100	6
MLP-like protein 328	*A. thaliana*	21542149	9E-81	6
Ubiquitin	*A. thaliana*	28202244	4E-31	6
60S ribosomal protein	*A. thaliana*	3123264	5E-30	5
glyoxalase II	*A. thaliana*	3913733	2E-67	5
zinc finger (C3HC4-type RING finger) family protein	*A. thaliana*	18403707	1E-45	5
60S ribosomal protein L5-1	*A. thaliana*	108860938	1E-22	4
60S ribosomal protein L8-3	*A. thaliana*	75221276	1E-35	4
cysteine proteinase	*A. thaliana*	1172872	2E-33	4
elongation factor 2	*B. vulgaris*	6015065	9E-70	4
Enolase	*A. thaliana*	119350	1E-53	4
galactinol-sucrose galactosyltransferase 2	*A. thaliana*	269969644	3E-126	4
protein kinase	*A. thaliana*	22327668	6E-142	4
S-adenosyl-L-homocystein hydrolase 1	*A. thaliana*	6174970	2E-101	4
secologanin synthase	*C. roseus*	461812	7E-65	4
rab family GTPase	*S. moellendorffii*	302787058	2E-51	4
ubiquitin-conjugating enzyme	*S. lycopersicum*	464981	6E-71	4
ω-6 fatty acid desaturase	*B. juncea*	3334184	1E-68	4

Supplementary Table S2. Selected examples of ESTs for genes related to cell wall metabolism

Gene annotation	Reference organism	GI number	E-value	ESTs
alpha-L-arabinofuranosidase 1	*A. thaliana*	75265802	5E-105	1
arabinogalactan peptidase 12	*A. thaliana*	75273536	2E-13	1
cellulose synthase-like protein A9	*A. thaliana*	75181330	1E-58	1
endoglucanase 5	*A. thaliana*	75186663	1E-32	1
endoglucanase 7	*A. thaliana*	75218974	8E-97	1
expansin A17	*A. thaliana*	20138096	3E-56	1
extensin 3	*A. thaliana*	21263622	3E-4	1
fasciclin-like arabinogalactan protein 2	*A. thaliana*	75207770	1E-42	1
fasciclin-like arabinogalactan protein 16	*A. thaliana*	75158667	3E-75	1
pectinesterase inhibitor 51	*A. thaliana*	75180831	3E-85	1
Polygalacturonase	*V. vinifera*	223635599	2E-49	1
polygalacturonase inhibitor 1	*A. thaliana*	21263838	4E-48	3
rhamnose biosynthetic enzyme 1	*A. thaliana*	62901057	6E-71	1
xyloglucan endotransglucosylase/hydrolase protein 14, XTH14	*A. thaliana*	38605534	3E-88	1
xyloglucan endotransglucosylase/hydrolase protein 26, XTH26	*A. thaliana*	38605519	3E-56	1

Supplementary Table S3. Selected examples of ESTs for genes related to transporter

Gene annotation	Reference organism	GI number	E-value	ESTs
ABC transporter signature motif	*A. lyrata subsp. lyrata*	297840569	1E-26	1
ABC-type transport-like protein	*A. thaliana*	4741190	5E-34	2
ADNT1, Adenine nucleotide transporter 1	*A. thaliana*	15234063	1E-55	2
amino acid transmembrane transporter, LHT1	*A. thaliana*	30693663	1E-100	1
amino acid transmembrane transporter, LHT1	*A. thaliana*	30693663	1E-100	1
Aquaporin	*B. oleracea*	11119335	3E-76	1
ATP:ADP antiporter	*A. thaliana*	15240640	2E-70	1
auxin resistant 1 protein	*B. rapa subsp. Campestris*	270272090	1E-47	1
cationic amino acid transporter 2, CAT2	*A. thaliana*	30696198	3E-23	1
efflux carrier, pin3	*B. juncea*	15485155	3E-96	1
epsilon-adaptin	*A. thaliana*	30692596	1E-29	1
ethylene insensitive 2, EIN2	*A. thaliana*	15242709	4E-86	1
ferric reducatse defective 3, antiporter	*A. thaliana*	15231918	3E-56	1
F-type proton ATPase	*A. thaliana*	166746	1E-74	3
glucose transporter	*A. thaliana*	21618276	2E-142	1
glycerol-3-phosphate transporter	*A. thaliana*	18408421	2E-134	1
GTP binding / phospholipase activator	*A. thaliana*	15228723	2E-81	1
GTP-binding protein	*A. thaliana*	3334322	4E-42	1
inorganic phosphate transporter 1-7 /Pi cotransporter	*A. lyrata subsp. lyrata*	297820224	4E-25	1
lipid transfer protein	*B. rapa subsp. Pekinensis*	48093506	3E-21	1
lipid transfer protein precursor	*B. rapa subsp. pekinensis*	122939101	8E-22	1
MATE efflux family protein	*A. thaliana*	15223402	3E-96	1
mitochondrial import receptor subunit TOM20	*A. lyrata subsp. lyrata*	297851168	8E-64	1
mitochondrial phosphate transporter	*A. lyrata subsp. lyrata*	297811523	4E-95	2
nitrate transporter	*B. napus*	9971067	3E-104	1

nitrate transporter	*A. lyrata subsp. lyrata*	297825869	8E-53	1
nitrate transporter	*B. napus*	9971067	6E-76	1
Nonclathrin coat protein, protein transporter	*A. thaliana*	110741159	1E-42	2
oligopeptide transporter	*A. thaliana*	145359208	8E-97	1
phosphate transporter	*A. lyrata subsp. lyrata*	297823783	2E-81	2
plant uncoupling mitochondrial protein 1, PUMP1	*A. thaliana*	15232420	4E-98	1
potassium channel	*A. thaliana*	79326141	3E-22	1
protein transporter	*A. thaliana*	238479693	1E-102	1
protein transporter	*A. thaliana*	79494763	2E-109	1
protein transporter	*Z. may*	226495857	7E-47	1
proton-dependent oligopeptide transport, POT	*A. thaliana*	15221883	2E-128	1
P-type proton ATPase	*A. thaliana*	62321204	4E-68	1
pyrophosphate-energized vacuolar membrane proton pump	*T. salsuginea*	60476796	4E-111	2
rab family GTPase	*S. moellendorffii*	302787058	2E-51	4
Rab GTPase	*A. lyrata subsp. Lyrata*	297830542	2E-81	1
transport protein particle; TRAPP	*A. thaliana*	15239731	4E-96	1
Transporter	*A. thaliana*	15234794	8E-57	1
two-pore calcium channel	*A. thaliana*	14041819	7E-68	1
vesicle-mediated transport	*A. thaliana*	30681419	6E-57	1
V-type proton ATPase	*R.communis*	255539062	2E-65	2
V-type proton ATPase	*A. thaliana*	15233060	4E-79	7
V-type proton ATPase	*E. salsugineum*	60476796	3E-134	2
V-type proton ATPase	*G. hirsutum*	111154399	7E-34	1
water channel-like protein	*A. thaliana*	21593526	5E-32	1
WD-40 repeat family protein, protein transport	*A. thaliana*	30695804	1E-51	1

Supplementary Table S4. Selected examples of ESTs for genes related to phytohormones biosynthesis and response

Gene annotation	Reference organism	GI number	E-value	ESTs
**Abscisic acid:**				
ABI5-binding protein 1	*A. thaliana*	75180099	2E-16	1
abscisic acid receptor PYL8	*A. thaliana*	75170450	5E-21	1
**Auxin:**				
26S proteasome subunit 4-like protein	*B. napus*	11045086	2E-102	1
26S proteasome AAA-ATPase subunit RPT4a	*A. thaliana*	6652884	3E-62	1
26S proteasome regulatory complex subunit subunit P42d	*A. thaliana*	1521950 3	9E-61	1
ARF1-binding protein	*A. thaliana*	62319903	5E-107	1
auxin-responsive family protein	*A. lyrata subsp. Lyrata*	21592781	2E-100	1
auxin-regulated protein	*P. trichocarpa*	109676318	6E-5	1
auxin resistant 1, AUX1	*A. thaliana*	18404642	2E-47	1
auxin transport protein, BIG	*A. thaliana*	21779966	2E-41	1
cullin-associated and neddylation dissociated 1, CAND1	*A. thaliana*	22325430	7E-51	1
E2 ubiquitin-conjugating enzyme, UBC 10	*A. thaliana*	183013548	2E-59	1
efflux carrier, pin3	*B. juncea*	15485155	3E-96	1
F-box/LRR-repeat protein 15	*A. thaliana*	124007179	9E-110	1
glutathione S-transferase parC	*N. tabacum*	1346215	2E-75	10
IAA7-auxin-response AUX/IAA family member	*Z. may*	195635917	1E-38	1
IAA-conjugate-resistant 4	*A. lyrata subsp. lyrata*	297850898	4E-101	1
nodulin-like protein	*A. thaliana*	5262203	2E-122	1
phytochrome-associated protein 1	*A. thaliana*	4093155	3E-78	3
pyrophosphate-energized vacuolar membrane proton pump	*T.salsuginea*	60476796	3E-111	2
secologanin synthase	*C. roseus*	461812	7E-65	4
ubiquitinating enzyme	*A. thaliana*	66354424	9E-71	1
ubiquitin conjugating enzyme 9	*A. thaliana*	18417097	1E-70	1
V-type proton ATPase	*A. thaliana*	62321641	9E-66	1
WD-40 repeat protein	*A. thaliana*	2289095	5E-56	1

**Brassinosteriod:**				
brassinazole-resistant 2, BIN2	*A. thaliana*	57012618	1E-17	2
BRI-EMS-suppressor 1, BES1	*A. thaliana*	42571545	3E-72	2
**Ethylene:**				
AP2-domain protein RAP2.2	*A. thaliana*	2281629	3E-50	1
ethylene insensitive 2, EIN2	*A. thaliana*	15242709	4E-86	1
ethylene-responsive transcription factor	*A. thaliana*	75264007	8E-15	1
S-adenosylmethionine synthase 3	*B. juncea*	75306464	1E-87	2
S-adenosylmethionine synthase 4	*B. juncea*	75306463	1E-98	1

Supplementary Table S5. Selected examples of ESTs for genes related to root development

**Gene annotation**	**Reference organism**	**GI number**	**E-value**	**ESTs**
auxin-responsive family protein	*A. lyrata subsp.*	21592781	2E-100	1
cullin-associated and neddylation dissociated 1, CAND1	*A. thaliana*	22325430	7E-51	1
ferredoxin-NADP reductase 2	*A. thaliana*	297851496	1E-56	1
nodulin-like protein	*A. thaliana*	5262203	2E-122	1
root hair defective 4, RHD4	*A. thaliana*	30693470	2E-99	1

Supplementary Table S6. Selected examples of ESTs for genes related to amino acid metabolism

Gene annotation	Reference organism	GI number	E-value	ESTs
**Arginine**				
arginine decarboxylase	*A. thaliana*	12229960	1E-113	6
**Aspartate**				
aspartate aminotransferase	*A. thaliana*	21542386	6E-32	1
**Cysteine and methionine**				
5-methyltetrahydropteroyltriglutamate--homocysteine methyltransferase	*A. thaliana*	8134566	2E-109	1
S-adenosyl-L-homocystein hydrolase 1	*A. thaliana*	6174970	2E-101	4
cysteine synthase	*A. thaliana*	11131561	2E-51	1
S-adenosylmethionine decarboxylase proenzyme 3	*A. thaliana*	15213968	2E-130	1
**Glycine, serine and threonine**				
D-3-phosphoglycerate dehydrogenase	*A. thaliana*	3122858	4E-62	1
diaminopimelate decarboxylase 1	*A. thaliana*	75306310	2E-88	1
glutamate synthase 1	*A. thaliana*	300680981	2E-37	6
**Histidine**				
histidine decarboxylase	*S. lycopersicum*	1706319	6E-72	1
**Phenylalanine**				
trans-cinnamate 4-monooxygenase	*A. thaliana*	3915085	2E-78	1
**Tryptophan**				
tryptophan N-hydroxylase 1	*A. thaliana*	12644083	4E-143	1
tryptophan synthase beta chain 2	*A. thaliana*	174779	5E-157	2

Supplemental Table 7. Selected examples of ESTs for genes related to stress response

Gene annotation	Reference organism	GI number	E-value	ESTs
alcohol dehydrogenase	*B. oleracea*	6684350	9E-73	1
aquaporin PIP1-5	*A. thaliana*	32363338	2E-33	2
Catalase	*B. napus*	5487875	2E-118	1
chaperone protein dnaJ 2	*A. thaliana*	21431768	3E-94	1
constitutively activated cell death 1, CAD1	*A. thaliana*	18397204	2E-55	1
copper/zinc superoxide dismutase	*A. lyrata subsp. Lyrata*	297826125	4E-81	1
dehydration-responsive family protein	*A. thaliana*	18405149	3E-64	2
dehydration stress-induced protein	*A. thaliana*	21553555	1E-66	1
dehydroascorbate reductase	*B. rapa subsp. Pekinensis*	33285914	2E-47	1
disease resistance response protein	*B. rapa*	157849646	1E-44	1
glutathione S-transferase 6	*A. thaliana*	20197312	1E-41	1
glutathione S-transferase parC	*N. tabacum*	1346215	5E-44	10
heat shock factor binding protein 3, HSFB3	*A. lyrata subsp. Lyrata*	297824131	9E-22	2
heat shock factor binding protein	*O. sativa japonica*	11862947	5E-20	1
heat shock protein 70	*A. thaliana*	240254411	2E-52	1
manganese superoxide dismutase 1	*B. napus*	169244541	1E-99	2
monodehydroascorbate reductase	*B. juncea*	4704613	3E-59	1
monodehydroascorbate reductase	*B. rapa subsp. Pekinensis*	14764532	1E-97	2
osmotin-like protein	*A. thaliana*	21542444	2E-41	3
Peroxidase	*A. thaliana*	15234648	2E-96	1
Peroxidase	*R. sativus var. niger*	166198115	2E-62	2
Peroxidase	*R. sativus var. niger*	49609452	3E-82	1
Peroxidase	*A. thaliana*	15237613	3E-87	2
Peroxidase	*A. thaliana*	2894574	5E-104	1
phosphate starvation-response 3.1, PYK10	*A. thaliana*	15232626	2E-90	1
senescence-associated cysteine protease	*B. oleracea*	18141289	5E-110	1
sulfite oxidase, SOX	*A. thaliana*	79295451	7E-110	1

superoxide dismutase	*R. sativus*	3114705	1E-15	1
thioredoxin H-type	*B. rapa*	11135129	9E-62	1
universal stress protein A-like protein	*A. thaliana*	115502895	1E-79	1
WD-40 repeat family protein, protein transport	*A. thaliana*	30695804	1E-51	1

Supplementary Table S8. Selected examples of ESTs for genes related to signal transduction

Gene annotation	Reference organism	GI number	E-value	ESTs
Transcription factor				
AP2-domain protein	*A. thaliana*	15219839	3E-14	1
AP2-domain protein RAP2.2	*A. thaliana*	2281629	3E-50	1
ARF1-binding protein	*A. thaliana*	62319903	2E-107	1
brassinosteroid-responsive ring-H2, BRH1	*A. thaliana*	15233117	5E-87	1
BRI1-EMS-Suppressor 1	*A. thaliana*	42571545	3E-72	2
flowering locus C1 variant 2	*B. rapa var. purpuraria*	282721250	1E-76	1
G-box binding factor 5	*A. lyrata subsp. lyrata*	297836540	5E-39	1
general regulatory factor 8	*A. thaliana*	30698122	2E-40	1
HAT22	*A. thaliana*	15235712	3E-106	1
heat shock factor binding protein	*O. sativa japonica*	11862947	5E-2	1
heat shock factor binding protein 3, HSFB3	*A. lyrata subsp. Lyrata*	297824131	9E-22	2
homeodomain protein KNAT1/BP	*A. thaliana*	19908861	1E-51	1
Myb-domain protein	*A. thaliana*	18395724	1E-56	2
Myb-domain protein 13	*A. thaliana*	15221419	1E-21	1
Myb-domain protein 51-2	*B. rapa subsp. Pekinensis*	238625815	5E-130	1
NAC-domain protein 14	*B. napus (rape)*	31322580	2E-96	1
NAC-domain protein 3	*B. napus*	31322568	2E-40	1
NAC-domain protein 58	*A. thaliana*	15229637	5E-51	1
PHD finger family protein	*A. lyrata subsp. Lyrata*	297849860	1E-27	1
reduced vernalization 1 response 1, VRN1	*A. thaliana*	30685150	8E-71	1
TOE2	*A. thaliana*	30697332	5E-6	1
WRKY-domain protein 21	*A. lyrata subsp. Lyrata*	297822775	1E-59	1
WRKY-domain protein 21-1	*B. napus*	206574950	6E-12	1
WRKY-domain protein 65-1	*B. napus (rape)*	206574990	2E-33	1
zinc finger (A20 and AN1) family protein	*B. rapa*	119720772	1E-63	1

zinc finger (C3HC4-type RING finger) family protein	*A. thaliana*	18403707	1E-45	5
zinc finger (CCCH-type) family protein	*A. thaliana*	15231090	3E-82	1
zinc finger (RanBP2-type) family protein	*A. thaliana*	34395889	4E-19	1
zinc finger family protein	*A. lyrata subsp. Lyrata*	297812437	5E-23	3
zinc finger family protein, salt inducible	*A. thaliana*	18410398	3E-45	1
**Calcium-related protein**				
calcium-binding EF hand family protein	*A. lyrata subsp.*	297848776	8E-109	1
calcium-binding protein, annexin 3	*B. juncea*	229458366	1E-68	1
calcium-dependent protein kinase 6	*A. lyrata subsp*	297799626	3E-127	1
calmodulin-binding adenosine diphosphatase	*A. lyrata subsp. Lyrata*	297811997	9E-60	1
calmodulin-binding channel, ATCNGC9	*A. thaliana*	15234769	5E-93	1
calmodulin-binding protein, IQD13	*A. thaliana*	15231733	2E-97	1
calmodulin-binding protein, IQD2	*A. thaliana*	145357576	2E-70	1
CBL-interacting protein kinase	*B. napus*	17224924	1E-89	2
CBL-interacting protein kinase 1	*A. lyrata subsp.*	297834664	9E-46	1
two-pore calcium channel, ATPC1	*A. thaliana*	18412295	1E-68	1
**Kinase**				
adenosine kinase 1, ADK1	*A. thaliana*	42572347	2E-18	1
calcium-dependent protein kinase 6	*A. lyrata subsp. Lyrata*	297799626	3E-127	1
CBL-interacting protein kinase	*Brassica napus (rape)*	17224924	1E-87	2
CBL-interacting protein kinase 1	*A. lyrata subsp.*	297834664	9E-46	1
chloroplastic adenylate kinase 1	*A. lyrata subsp. Lyrata*	297823551	2E-85	1
cyclin-dependent kinase C1	*A. lyrata subsp. Lyrata*	297811129	1E-92	1
lectin protein kinase	*A. lyrata subsp. Lyrata*	297802800	2E-76	1
leucine-rich repeat protein kinase	*A. thaliana*	18422906	1E-86	1
light repressible receptor protein kinase	*A. thaliana*	1321686	3E-79	1
pfkB-type carbohydrate kinase	*A. lyrata subsp.*	297844934	2E-62	1

	Lyrata			
phosphatidylinositol-4-phosphate 5-kinase,	*A. lyrata subsp. Lyrata*	297845202	7E-100	1
phosphoenolpyruvate carboxykinase 1, PCK1	*A. thaliana*	15235730	2E-65	2
protein kinase	*A. lyrata subsp. Lyrata*	297797331	5E-54	3
protein kinase	*A. thaliana*	22327668	6E-142	4
protein kinase, D6PK	*A. thaliana*	15241076	2E-113	1
protein kinase, FUS3	*A. lyrata subsp. Lyrata*	297820088	8E-82	1
protein kinase, WNK4	*A. thaliana*	15237174	3E-91	1
pyruvate kinase	*A. thaliana*	110739133	4E-49	2
receptor protein kinase, CLAVATA1 precursor	*A. thaliana*	15239123	4E-50	2
receptor-associated kinase, SUB	*A. thaliana*	30682076	5E-63	1
ribitol kinase	*A. thaliana*	42567264	7E-108	1
shaggy related protein kinase, ASK-γ	*A. thaliana*	21553877	4E-165	2
**Phosphatase**				
3(2),5-bisphosphate nucleotidase	*A. thaliana*	21618152	3E-30	1
calcium-binding protein, ABI1	*A. thaliana*	509419	9E-54	1
calmodulin-binding adenosine diphosphatase	*A. lyrata subsp. Lyrata*	297811997	9E-60	1
phosphatidylinositol-4,5-bisphosphate 5-phosphatase	*A. thaliana*	30693470	9E-100	1
phospholipase D α1	*Brassica oleracea var. capitata*	13124444	4E-110	1
protein phosphatase 2A	*A. thaliana*	683502	8E-133	1
protein phosphatase 2C	*Thellungiella halophila*	20340237	3E-60	1
trehalose-6-phosphate phosphatase	*A. thaliana*	15235713	1E-68	1
type I inositol polyphosphate 5-phosphatase	*A. thaliana*	42569699	9E-57	1
type I phosphodiesterase/nucleotide pyrophosphatase family protein	*A. lyrata subsp. Lyrata*	297799022	3E-115	1
vacuolar purple acid phosphatase, AtPAP26	*A. lyrata subsp. Lyrata*	297805048	2E-75	1

Supplementary Table S9. Selected examples of ESTs for genes related to cell structure and division

Gene annotation	Reference organism	GI number	E-value	ESTs
tubulin alpha-1, TUA1	*G. hirsutum*	54036491	5E-22	1
tubulin alpha-1, TUA1	*A. thaliana*	267070	3E-88	1
tubulin beta-3, TUB3	*G. hirsutum*	54036491	3E-67	1
tubulin beta-5, TUB5	*T. aestivum*	8928427	7E-63	2
tubulin beta-8, TUB8	*A. thaliana*	2773526	2E-132	1
cell division protease ftsH homolog 10, AtftsH10	*A. thaliana*	75331189	9E-49	2
cell division control protein 48 homolog A, AtCDC48a	*A. thaliana*	1705677	2E-157	1
WUS-interacting protein 2	*A. thaliana*	298352695	3E-75	1

Supplemental Table S10: List of Primers

	**forward sequence 5'-3'**	**Reverse sequence 3'-5'**
>Phytochrome associated protein 1	GACCTCTGAAGAGTTGGTCTACGGCA	TGGCCTCCGGTTCGTTCGTTC
>polygalacturonase inhibitor	TAACCACCGCGTCACCGCCCTA	CCGGTCAGATTCGTCCAGCTG
>Rab family Gtpase	CATTGCCCCTCGGTAATAAGCAGTG	CCTTATCGGTGACAGCGGTGTTGG
>V-typeATPAse	GCACCCCAGGCTTTACACTTT	GCGTTGGATGCATAGCTTGA
>Aquaporin	CTCCCACGAACGGTCCAACCC	TTACAGCACCGGGACTGCTCTTGG
>ARF1-binding protein	CCGAGGTCAACCACGAAGGCAT	GTTCCAGTTGAAATAGCGTGCCAGG
>Expansin	CCGGGTTAATGGTTTTTTTTCC	CTGCTTGAGTCTTCCCCAGATT
>tryptophan n-hydroxylase 1	GTTAAAGGCGGCGACAGGATGGA	GGCAACCCATTGCTTACCGCTG
>ATCNGC9	CATCAAACGCCATCTCTGTCTGGC	CCACTTCTCCCACCGTCTGTCGT
>inorganic phosphate transporter 1-4	CTTCACCGACGCTTACGATCTCTTCT	CGACAGAGCAAAGGACCATGACCA
>tubulin beta-5	GTGGACGAACAGATGATAAACGTGCAG	TCCTGAACATGGCTGTGAACTGCTC
>topless-related protein 4-like	TCATCAGAACAGGGTAACAGGTCTTGCT	CGAACCCACTGCATCCACCAATC
>Secologanin synthase	AGGATAAGATGAGCACCAAACTGTGGG	AACGATGCAGGGGAGTTCAAGCC
>epidermis glycoprotein	AGCCGTGGAAGATTCCGACACC	ATTCTACACTCGTCGTTACCGTCGGTAT
>Arginine decarboxylase	AAGCTGAAGCAGAGATGTGTTGAAGGTT	CTTCCCGTCACTATCACACGTCAAATCC
>Cell division protease ftsh	GGTCACGCGGTTGCTGGTTGGT	CAATACCTGTTCGGCTGCACGG
>Aux1	GCTGCGCACGCTCCGGTCTCC-3	TGAGAGCTTAACACGCATTCAAAGGGGAT
>efflux carrier pin 3	TCTGATGCTGTTGATGGCTATGT	CTGAGCCAGGGAGCTGACA
>RHD4	AACGCGTTGGATGCATAGC	CCCCAGGCTTTACACTTTATGCT
>Glutathione s-transferase ParC	CGCTGCCCTATAGTGAGTCGTA	TGAGCGGATAACAATTTCACACA
**Internal control**		
BnActin	GTGACAATGGAACTGGAATGG	ACGGAGGATAGCGTGAGGAA
